# Working with my brain and not against it

**DOI:** 10.7554/eLife.95068

**Published:** 2023-12-11

**Authors:** Tigist Tamir

**Affiliations:** 1 Koch Institute for Integrative Cancer Research at MIT Cambridge United States

**Keywords:** sparks of change, being neurodivergent in academia, research culture, ADHD

## Abstract

When attempts to capitalize on her undiagnosed ADHD traits led to repeated cycles of overwork and burnout, a postdoc re-evaluated how she faces the daily challenges of being a neurodivergent scientist.

When I was initially diagnosed with depression during my PhD, neither my healthcare provider nor I dug deeper into the why. It was only six years later, during my postdoc, that “neurodiversity” entered my vocabulary. Suddenly there were words that could explain my lived experience, and I finally realized that my mental health challenges could be traced back to unaddressed ADHD, or “attention deficit hyperactivity disorder”. For years I had entered constant cycles of overwork and burnout which had driven me to chronic depression. To make it through my training as a scientist, I had become adept at masking my neurodivergent traits and mirroring the behavior of those around me; it took me the better part of a decade to even realize how mentally draining it was to create this “normal” version of myself.

Born and raised in Ethiopia until my teenage years, I had felt from a young age that education was a great equalizer, both essential and a privilege. I started university in the United States as an excellent student whose self-image was tied to her academic achievements. For a long time, I assumed that because I had previously done so well in education that I couldn’t possibly be struggling with ADHD. Only now can I see that high school had offered me the structure and small class sizes I needed to fulfill my potential, not to mention the largely unrestricted time before and after classes to pester my teachers with questions and catch up on studying.

All of this disappeared at university. Lost in a sea of students, my barely existent organizational skills were no match for the sudden complexity of daily tasks. I bought planner after planner, all of them ending up barely used in a pile (if only I had realized my challenges weren’t going to disappear because of some magical notebooks!). It didn’t matter that I spent long hours studying or desperately crammed at 2am for an 8am exam, I couldn’t grasp enough of the material to do well in class. It was so frustrating.

It also didn’t help that I was constantly questioning if I belonged because I didn’t see many students who looked like me in my classes. I was unsure of how to navigate the academic space and assumed I was supposed to make it on my own. I can count on one hand the number of times I went to office hours because I didn’t know what to ask or didn’t want to feel stupid. The few times I mustered enough confidence to seek support, I just ended up feeling worse due to experiencing microagressions from some of my professors. Ultimately, my self-confidence shattered – until, at the end of my first year, a teaching assistant recognized that I had a knack for research and allowed me to work on their project.

Something clicked. Staring for hours through the confocal microscope, I felt nothing but utter joy. Research offered the constant novelty I had craved in lecture halls, each failed experiment an exciting intellectual puzzle. In the lab, my non-linear thinking process, my ability to hyperfocus, my need for novelty were assets and not flaws. I was relieved to have finally found something I was good at, a place for me to continue in science where I could lean into my strengths and have supportive mentors.

When I got accepted into my PhD program in North Carolina, I thought I’d need to give it my all. I remembered the speech that most minorities get: “You have to work twice as hard and be exceptional to compete in this unfair world.” I felt an immense pressure to not squander this privilege, this opportunity. While my curiosity gave me drive, my upbringing provided me with a sense of purpose and responsibility. Those are great qualities, in moderation; when they take too much space, little room is left for us to understand ourselves. It’s only since my ADHD diagnosis that I’ve taken the time to look back and reflect.

I now see that I actively tried to harness my ADHD traits for my own advantage during my PhD and, for a while, it seemed to work. I used my impulsive nature to take on big new projects and persisted to bring them to fruition. I embraced teamwork as a form of “body doubling” (i.e., working with someone else present to boost my focus and productivity by sharing motivation and accountability) and set up collaborations that proved mutually beneficial. I would, however, end up planning way too many experiments for a reasonable day because my executive dysfunction was often out of control and my time blindness made me unable to gauge how long a task would take. These traits would also cause me to just keep working until hunger or exhaustion physically stopped me. Yet the moments of great creativity that helped move my project forward made it easy for me to often dismiss these daily challenges.

I was then working so much that I never really replenished my energy stocks with enough rest. In typical ADHD fashion, I even turned Tae Kwon Do, the hobby I had taken up to balance my physical and social well-being, into an obsession and began entering too many competitions. Unfortunate microaggressions with racist undertones further added to my mental fatigue, from the subtle “you speak so well” to the campus police deciding they should randomly check my ID while I was clearly working at my desk. It all took its toll, and, towards the latter half of my PhD, I had a hard time coping and felt that I should quit.

My cultural background hadn’t put much weight on mental health or different ways of thinking. When I was told I had depression, I refused to accept the diagnosis at first because it made me feel like I was weak. As a minority in academia, I thought I couldn’t afford to show any flaw, so I became very guarded and isolated myself from my peers. It was only when my physical health collapsed too that I was forced to have an open conversation with my mentors. They reassured me that I wouldn’t lose out on my dream because of mental health challenges. With their support, I started going to therapy and finished my PhD. Perhaps this story would have ended here if it hadn’t been for the pandemic lockdown hitting a month into my postdoc and driving me through a dark phase. While receiving treatment, this time I received an extended professional assessment which led to my ADHD diagnosis. I felt such relief; sometimes, naming something is half the battle.

I started educating myself on how my brain works and once I got to a point of acceptance, I realized that I could find new strategies for myself. I sought out treatments that helped me reset my brain and I started to re-evaluate how to tackle the daily challenges of being a scientist who has ADHD (see Box 1). Even simple adjustments, such as finding optimal times to read, write and do experiments, or paying attention to feelings of anxiety, have moved me away from the constant hopelessness. I now have learned to prioritize rest and find ways to spark joy in my daily routine because I am working with my brain and not against it.

Box 1. **Tactics that have helped me as a scientist who has ADHD.**

- Aligning my tasks with my energy levels throughout the day and repurposing my hyperactivity. Mornings are when I'm most motivated and are thus dedicated to challenging tasks like writing or paperwork. Afternoons are reserved for more routine or enjoyable activities. When I feel hyperactive, I do lab jobs like making buffers, organizing freezer spaces, restocking supplies.
- Using Pomodoro timers to break my work into 25-minute chunks interspersed with short breaks. This has also helped me tackle time blindness and keep track of how long tasks really take. It also stops me from starting experiments when there isn’t enough time, enabling me to leave at a reasonable hour.
- Joining a virtual ADHD co-working group that also uses the Pomodoro method. This has significantly boosted my productivity by fostering shared focus and accountability and by reducing distractions during desk tasks like writing and data analysis.
- Switching from planners to a digital note-taking system. I now use a reMarkable notebook, back up notes as PDFs on my Google Drive, and integrate appointments and blocks of time for regular tasks into my online calendar so that I don’t forget them.
- Embracing AI tools as they reduce the burden on my working memory. For example, I use Goblin.tools to make task lists and break down tasks into smaller steps, and ResearchRabbit to streamline literature searches and keep up to date with my field.
- Joining peer support groups. I am fortunate to both support and be supported by peers through Leading Edge and the National Black Postdoctoral Association (NBPA). For example, in the NBPA we specifically support neurodivergent members identify useful resources and strategies, and to have open discussions. The NBPA has also developed an Emergency Support Program for trainees who need someone to talk to during a crisis.

I’m learning that some ADHD-friendly strategies won’t work for me, or not at this time, and that I’ll sometimes have to adjust existing systems built without neurodivergence in mind. I try not to let this bother me as much anymore. Don’t get me wrong, I have bad days where none of my coping skills or ADHD strategies help. But now I know to give myself the grace and compassion I need to do a better job the next day. I am still a work in progress, and I have so much more to learn and to unlearn.

I have also learned from experience that both mentors and mentees can benefit from more resources to support a healthy work environment. As academics I feel we should strive to create an environment that centers compassion and understanding. We don’t stop being human when we step into the lab, so we shouldn’t shy away from aspects of our humanity either.

Lastly, one of the best things I’ve done for myself was to find places where I can ask for advice and find people to relate to. Being a member of the National Black Postdoctoral Association, for example, has allowed me to find peers who understand the unique challenges of Black scholars, including those who are neurodivergent like me. With support from my mentors and peers, it has been easier to bring my authentic self to the lab.

## About this article

This Sparks of Change article is part of a series of articles on being neurodivergent in academia, which includes a list of tips, resources and tools collated by neurodivergent scientists.

